# The value of applying a melatonin antagonist (Luzindole) in improving the success rate of the bipedal rat scoliosis model

**DOI:** 10.1186/s12891-017-1500-x

**Published:** 2017-04-04

**Authors:** Shuo Yang, Chaojun Zheng, Jianyuan Jiang, Feizhou Lu, Xinlei Xia, Wei Zhu, Xiang Jin, Xiaosheng Ma

**Affiliations:** 1 Department of Orthopedics, Huashan Hospital, Fudan University, No.12, Middle Wulumuqi Road, Shanghai, 200040 China; 2 Department of Orthopedics, The Fifth People’s Hospital, Fudan University, No.128, Ruili Road, Shanghai, 200240 China

**Keywords:** Scoliosis, Melatonin antagonist, Bipedal rat model

## Abstract

**Background:**

An ideal animal model has always been the key to research the pathogenesis and treatment of adolescent idiopathic scoliosis (AIS), while available methods have obvious disadvantages. The deficiency of melatonin has been proved relating to AIS. In this research, we intended to apply Luzindole, the melatonin antagonist, in bipedal rat model, for the block of combination of melatonin and its receptor, to inhibit the melatonin effect, and then to understand whether this method can effectively improve the scoliosis rate of bipedal rat model, and investigate the role of melatonin in scoliosis. To investigate the feasibility of improving the success rate of bipedal rat scoliosis model via intraperitoneal injection of melatonin antagonist (Luzindole).

**Methods:**

A total of 60 3-weeks-old Sprague-Dawley rats were included in this study, and were divided into 3 groups (A, B and C). Each group included 20 rats. Osteotomy of the bilateral proximal humerus and proximal tailbone was performed in group A and group B; intraperitoneal injection of Luzindole (0.2 mg/kg) was performed in group A and group C. X-rays were taken before the surgery, 1 month after the surgery, 3 months after the surgery, and 6 months after the surgery, to calculate the Cobb’s angle of the spine (>10° was considered scoliosis). The weight of every rat was also measured at the same time. Rats were euthanized 6 months after surgery to determine the calmodulin level in thrombocytes.

**Results:**

The rate of scoliosis in group A (14/20) was significantly higher than those in group B (6/20) and group C (0/20) (*P* < 0.05). The differences in the weights of the 3 groups were non-significant; as were differences in the calmodulin level in thrombocytes.

**Conclusion:**

The application of the melatonin antagonist of Luzindole can improve the success rate of the bipedal rat scoliosis model. Meanwhile, this study indicates that a decreased melatonin level is not the primary cause of scoliosis, but that it may increase the likelihood and severity of scoliosis.

## Background

Adolescent idiopathic scoliosis (AIS) is a three-dimensional spinal deformity that occurs around the puberty, though the cause is currently unclear [1, 2]. However, related randomized controlled trial (RCT) studies in humans are unethical [3]. Therefore, an ideal animal model has always been the key to researching the pathogenesis and treatment of AIS.

The bipedal rat model was established by Goff in 1957. In Goff’s study, osteotomy of the bilateral proximal humerus and proximal tailbone was performed to make bipedal rats, and scoliosis could develop due to continuous weight bearing. Its beneficial qualities, including ease of induction, low cost, and the similar anatomical construction of rats to human beings, led to the widespread use of the bipedal rat model in studies on AIS [4]. However, due to a low success rate, the establishment of the bipedal rat model requires additional factors to increase the success rate [3, 5–8].

In 1959, Thillard unintentionally discovered that after pineal body excision, chickens always develop scoliosis, which indicates that abnormality of the melatonin level has a certain correlation with scoliosis [9]. Recent studies have shown that AIS patients have a lower melatonin level compared to individuals of the same age group, which indicates that a lower melatonin level has an explicit correlation with AIS [10, 11]. Therefore, a bipedal rat model with melatonin inhibition has been one of the major methods used to establish a scoliosis animal model. However, commonly used methods, such as pineal body excision [12, 13], continuous illumination [11, 14], and C57BL mice [7], still have some disadvantages, including their high cost, complicated preparation, and high mortality rate, among others etc. Some studies have even proved that a decreased melatonin level alone cannot effectively induce scoliosis [5], perhaps because the blood melatonin level has been partially inhibited, but the effect of melatonin has not been impacted.

Therefore, in this study, we administered Luzindole, a melatonin antagonist that blocks melatonin binding to its receptor, in the bipedal rat model, in order to inhibit the effects of melatonin. We sought to understand whether this method can effectively increase the scoliosis rate of bipedal rat model, and to investigate the role of melatonin in scoliosis.

## Methods

### Experimental animals

This study was approved by the Ethic Committee of Fudan University, and all the procedures were completed in the Experimental Animals Department of Medical College of Fudan University. This study used 60 3-week-old Sprague-Dawley rats (SPF level), which were purchased from the Experimental Animals Department of the Medical College of Fudan University.

### Experimental method

The rats used for the experiment were randomly separated into 3 groups (A, B and C), with 20 included in each group. All the rats were kept in indoor in cages with a room temperature of 22 ± 2 °C. Natural light or light was available from 8:00 – 20:00, and the lights were off during the remaining hours. Groups A and B were fed in raised cages by a qualified person, with adequate food and water. The height at which food and water were placed was modified based on the growth rate of the rats. After 1 week of adaptive growth, groups A and B underwent osteotomy of the bilateral proximal humerus and proximal tailbone following anesthetization (10% chloral hydrate 3 ml/kg intraperitoneal injection) [4, 13]. Rats in Group A and Group C received an intraperitoneal injection of the melatonin antagonist (Luzindole) (sigma, USA) at 0.2 mg/kg [15].

Weight was measured before the surgery, 1 month after the surgery, 3 months after the surgery, and 6 months after the surgery. X-rays (41kv, 2.80mAs) of the full spine were taken to calculate the Cobb’s angle of the spine (>10° was considered scoliosis) at the same time. At the end of the experiment (6 months after the surgery), all rats were euthanized by overdose anesthetics, and thrombocytes were centrifugalized from venous blood to determine the calmodulin level by ELISA.

### Statistical methods

SPSS 12.0 was used for statistical analysis. The Kolmogorov-Smirnov test was used to determine whether the data of each group conformed to a Gaussian distribution. At each time point, the Kruskal-Wallis test was used to compare Cobb’s angle, weight, and thrombocyte calmodulin level between groups. The scoliosis rates of different groups were compared using the chi-square test. A *p* value < 0.05 was considered statistically significant.

## Results

One rat in Group A died as a result of anesthetization, 1 rat in Group B died of infection, and all other rats lived to the end of experiment in healthy condition (data in Table 1). Before the surgery, scoliosis or other deformity was not observed in any of the rats (Cobb’s angle: 2.7 ± 2.1°).

**Table 1:** Cobb’s angle of each group at different time

	Before surgery (°)	1 month after surgery (°)	3 months after surgery (°)	6 months after surgery (°)
Group A	2.8 ± 2.2 (20/20)	7.0 ± 5.4 (19/20)	13.0 ± 9.3 (19/20)	17.2 ± 11.6 (19/20)
Number of scoliosis	/	3	12	14
Group B	2.6 ± 2.0 (20/20)	5.0 ± 4.3 (19/20)	7.8 ± 8.0 (19/20)	9.1 ± 9.2 (19/20)
Number of scoliosis		1	3	6
Group C	2.6 ± 2.2 (20/20)	3.2 ± 2.6 (20/20)	3.6 ± 2.7 (20/20)	3.8 ± 2.9 (20/20)
Number of scoliosis	/	/	/	/

(a/b): b is the number of rats included in the group;a is the number of rats included in the measurement

“/”: No scoliosis

**Fig. 1:**
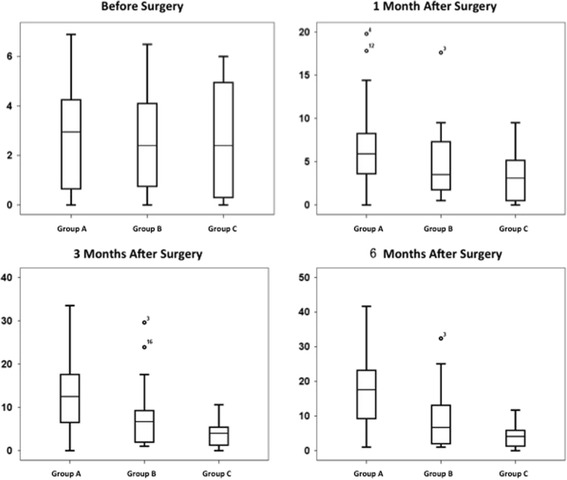
Cobb’s angles in each group of bipedal rats at different time. There were no significant differences between the Cobb’s angle of Group A and Group B at the time before surgery, 1 month after surgery and 3 months after surgery (*P* > 0.05); At the time of 1 month after surgery, 3 months after surgery, 6 months after surgery, the Cobb’s angle of both Group A and Group B was significantly bigger than that in Group C (*P* < 0.05)

**Fig. 2:**
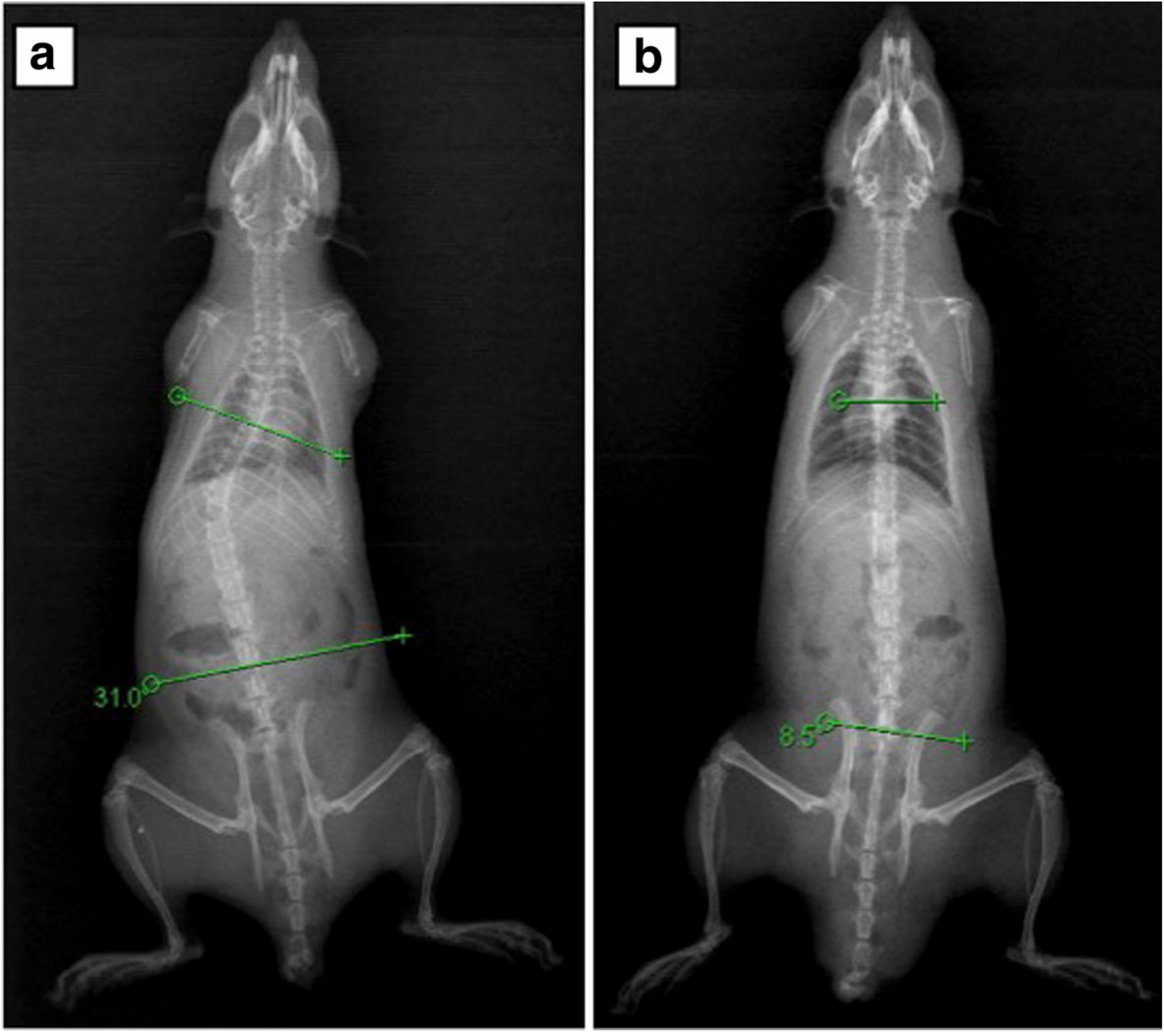
X-ray films of bipedal rats in Group A and Group B 6 months after surgery. The scoliosis was more severe in the Luzindole-injected bipedal rats

There were no significant differences in scoliosis rate or severity of scoliosis between Group A and Group B (*P* > 0.05) (Fig. 1) at 1 month after surgery. At 3 months after surgery, the scoliosis rate in Group A was significantly higher than that in Group B (*P* < 0.05), but the difference in the severity of scoliosis was non-significant (Fig. 1). At 6 months after surgery, the scoliosis rate in Group A was significantly higher than that in Group B (*P* < 0.05), and the Cobb’s angle of Group A was significantly greater than that of Group B (*P* < 0.05) (Fig. 1; Fig. 2). During the whole experiment, rats in Group C did not show any scoliosis, and the Cobb’s angle of Group C was significantly smaller than those of Group A and Group B at any time point (*P* < 0.05) (Table 1).

The differences of the calmodulin level in thrombocyte among three groups were non-significant (Group A: 140.0 ± 9.6; Group B: 137.4 ± 8.6; Group C: 138.4 ± 7.8).As such no significant difference was found in data of weight among three groups (Table 2) (*P* > 0.05).

**Table 2:** Weight of each group at different time

	Before surgery	1 month after surgery	3 months after surgery	6 months after surgery
Group A	100.7 ± 6.0 (20/20)	180.7 ± 24.4 (19/20)	290.0 ± 30.3 (19/20)	360.1 ± 26.5 (19/20)
Group B	97.5 ± 4.8 (20/20)	190.8 ± 26.6 (19/20)	283.5 ± 30.8 (19/20)	349.1 ± 24.7 (19/20)
Group C	97.5 ± 5.4 (20/20)	183.1 ± 27.1 (20/20)	289.6 ± 26.9 (20/20)	355.5 ± 22.9 (20/20)

(a/b): b is the number of rats included in the group;a is the number of rats included in the measurement

## Discussion

This study proves that the application of the melatonin antagonist Luzindole can improve the success rate of the bipedal rat scoliosis model.

Compared to the non-injection group, most rats in the Luzindole injection group developed scoliosis by 3 months after surgery. This confirmed that the decrease in melatonin level during early growth facilitated scoliosis or the tendency to develop scoliosis. With weight gain, scoliosis may be aggravated because of the biomechanical factors of imbalance. Additionally, no additional rats in Group A developed scoliosis after 3 months after surgery and the increase in Cobb’s angle was much greater than that in Group B.

However, in this study, we also showed that Luzindole injection alone could not induce scoliosis in rats, indicating that melatonin deficiency or inhibition is not sufficient to induce scoliosis. O’Kelly’s study showed that the fore feet rats did not develop scoliosis after pineal body excision [5], which indicates that the role of melatonin deficiency in the pathogenesis of scoliosis is mostly synergistic; Wu’s study has confirmed this finding [16].

In this study, the scoliosis rate in the Luzindole injection group was approximately 70%, and the remaining rats in the group did not show scoliosis at the end of the experiment. This may be attributable to a partial block of the melatonin receptor. Melatonin has two receptor subtypes in human body, MT1B and MT1A. Luzindole only blocks the high-affinity receptor, MT1B, while leaving the low-affinity receptor, MT1A, uninhibited [17]. This may impact the scoliosis rate. As such, there are two peaks in the melatonin level during the course of a day (12:00 and 18:00-20:00) [15, 16], and we only inhibited the second peak, while the first peak was not inhibited, which may have led to the low scoliosis rate. In addition, according to Benitez-King’s report, melatonin can act as an antagonist of calmodulin, and calmodulin can also play an important role in the pathogenesis of scoliosis [18]. Akel’s study proved that the application of a calmodulin antagonist can protect mice from scoliosis [19]. Therefore, after using Luzindole to block the melatonin receptor, there would be spare melatonin in the body, which may act as an antagonist of calmodulin to prevent scoliosis [20]. However, in this study, the calmodulin level did not show significant difference between the groups, which indicates that Luzindole was not overused. However, the most suitable dose remains to be investigated.

The small sample size is an additional limitation to the injection dose issue. More accurate results will require larger a sample size and a more refined group division. Meanwhile, the scoliosis rate may improve if Luzindole were injected at both daily melatonin peaks. This will need further study to determine.

## Conclusion

The application of the melatonin antagonist of Luzindole can improve the success rate of the bipedal rat scoliosis model. Meanwhile, this study indicates that a decreased melatonin level is not the primary cause of scoliosis, but that it may increase the likelihood and severity of scoliosis.

## Abbreviations

AIS: Adolescent idiopathic scoliosis
RCT: Randomized controlled trial
